# Animation and interactivity facilitate acquisition of pediatric life support skills: a randomized controlled trial using virtual patients versus video instruction

**DOI:** 10.1186/s12909-018-1442-5

**Published:** 2019-01-05

**Authors:** Ronny Lehmann, Thomas Lutz, Astrid Helling-Bakki, Sebastian Kummer, Sören Huwendiek, Hans Martin Bosse

**Affiliations:** 1 Department of General Pediatrics, Center for Pediatrics and Adolescent Medicine, University Hospital Heidelberg, Im Neuenheimer Feld 430, 69120 Heidelberg, Germany; 2 Department of General Pediatrics, Neonatology and Pediatric Cardiology, University Children’s Hospital, Heinrich-Heine-University, Moorenstr. 5, 40225 Düsseldorf, Germany; 3 University of Bern, Medical Faculty, Institute for Medical Education, Mittelstrasse 43, 3012 Bern, Switzerland

**Keywords:** Pediatric basic life support, Blended learning, Virtual patients, Video instruction, Performance rating

## Abstract

**Background:**

Several promising studies suggest a positive impact of interactive and media-enriched e-learning resources such as virtual patients (VP) on skill acquisition in pediatric basic life support (PBLS). This study investigates which immanent VP components account for this effect.

**Methods:**

*N* = 103 medical students in their 5th year were assigned to one of three groups: a video group prepared with self-instructional videos on PBLS (*N* = 37); an animation-enriched VP group with VP containing interactive questions (*N* = 35), static *and* animated media, and a static VP group with VP containing interactive questions and only static media (*N* = 31). Subsequent PBLS demonstrations were video-documented and scored for adherence to guideline-based algorithm, temporal demands (such as correct pace of rescue breaths and chest compressions), and quality of procedural steps (e.g., correct head positioning), as well as overall competency by two group-blinded, independent pediatricians.

**Results:**

Groups did not differ with regard to adherence to correct algorithm (88.7 ± 10.3, 93.3 ± 6.7 and 90.3 ± 10.5, respectively). Self-instruction with animated media – through videos or animation-enriched VP – resulted in a better adherence to temporal demands, as compared with training with static VP (64.5 ± 26.3 and 50.7 ± 25.7, respectively, vs. 23.8 ± 21.0). Procedural quality by the video group was slightly inferior compared with the animation-enriched VP group (79.5 ± 12.3 vs. 82.0 ± 11.9), and distinct inferior in overall ‘competent’ ratings (43.2% vs. 65.7%). The static VP group performed considerably most poorly of all three groups (temporal adherence 73.2 ± 11.9 and 19.4% ‘competent’ ratings).

**Conclusions:**

VP can feasibly enhance PBLS skill acquisition. Thoughtful design of animations *and* interactivity of the VP further improves such skill acquisition, both in quality of performance and in adherence to temporal demands.

## Background

Different approaches for improved dissemination of basic life support (BLS) and pediatric basic life support (PBLS) skills have been described. Traditionally, instructor-led courses use a 4-step approach for BLS teaching [1]. The instructional use of numerous multimedia and e-learning techniques has recently been the focus of educational research for dissemination of such resuscitation skills. Some of the video-based approaches described improved performance in cardiopulmonary resuscitation (CPR) after self-instruction compared with traditional classroom instruction [2, 3]. Animations, especially videos, are widely believed to facilitate procedural learning through dynamic and realistic presentation of learning content [4]. Cognitive effort is believed to be reduced when creating mental pictorial representation of content through guided, animated narration instead of static illustrations [5]. Also, an ‘interest effect’ has been described for authentic animations increasing learners’ motivation and cognitive engagement [5]. Note: For the purposes of this paper, we use the term animation (or animated media) to refer to any kind of motion picture including video with or without computer-generated supplements to confine it against the use of static media. Electronic teaching furthermore offers effective dissemination of resuscitation techniques through flexible access [6–9]. In this contex, the learner defines learning content, sequence, pace, and time to best meet educational needs [10]. Besides acquisition of knowledge, appropriate e-learning techniques foster acquisition of psychomotor skills and the development of attitudes [11]. Multimedia and e-learning have shown effectiveness in teaching BLS by providing self-paced, interactive learning environments [6, 8, 12–14]. E-learning courses have been reported as having equal outcomes in terms of CPR knowledge and performance compared with traditional instructor-led courses [7, 15, 16]. However, e-learning is most effective – in theory and practice – when enhancing instructor-led, individual feedback providing teaching formats which is referred to as ‘blended learning’ [9–11, 17].

Virtual patients (VP) are media-enriched e-learning resources that offer interaction with the learner [11, 18, 19]. Originally, they were developed to foster clinical reasoning and decision making skills [11, 18]. Within VP, corresponding animations can be provided along with a presented clinical case, e.g. with embedded video clips, and they offer guidance and feedback for taking care of the electronic case [11]. VP allow deliberate practice in a case-based environment with feedback beyond the mere presentation of learning content [18]. Thus, VP can provide an educational framework for the application of various educational strategies such as integration of animation for effort and interest effects, optimizing cognitive load (e.g., through spatial contiguity), and best complemented as blended learning activities [5, 10, 11, 20]. Studies on the use of VP for psychomotor skill acquisition are scarce, particularly when regarding these potential inhering benefits for complex procedures. Several promising reports are available on their effectiveness in basic life support-automated external defibrillator (BLS-AED) courses for both knowledge and skill acquisition [12, 21, 22]. Reder et al. showed measurably improved CPR skills when adding a hands-on training to a computer simulation, even though the electronic simulation alone was able to sufficiently teach AED skills [22]. Despite all positive reports on new instructional methods, comparisons of different methods are scarce. Past studies often did not include a control group (e.g. de Vries and Handley [12]) or compared intervention to no intervention (e.g. Kononowicz et al. [21]).

Our research group recently showed that a blended learning approach using VP not only improved decision making skills and procedural knowledge, it also improved acquired PBLS *hands-on skills* compared with a standard approach [23]. These effects were observed before and still after equivalent hands-on training in several procedural domains. We showed distinct improvements in the adherence to the correct guideline-based algorithm, to temporal demands deriving from the guidelines (such as appropriate pace in CPR) and to the quality of procedural steps (e.g., correct head positioning), as well as in distinctly improved ratings in overall competency [23].

It remains unclear which immanent VP components account for improving PBLS performance. In the present study, we elucidated the differential effects of interactivity and animations in VP, comparing them to the use of static-only media within VP and non-interactive video instruction.

## Methods

### Participants and curricular setting

During the winter term of 2016/2017, 5th-year medical students of the Medical School at the University of Heidelberg, Germany, were invited to participate in this study within their pediatric rotations. All students had already undergone CPR trainings in adults before within the medical curriculum. In the pediatric rotation, PBLS is part of the curriculum and is offered as blended learning with preparatory VP and subsequent skills laboratory training [24]. For this study, the preparatory phase was conducted as a presence session on stationary computers. Whereas preparation for the subsequent skills laboratory on PBLS was a mandatory part of the curriculum, participation in the study assessments was voluntary and data was collected anonymously. Written informed consent was obtained from all participants. The study was approved by the Ethics Commission of the Medical Faculty Heidelberg, Germany.

### Design

**Fig. 1:**
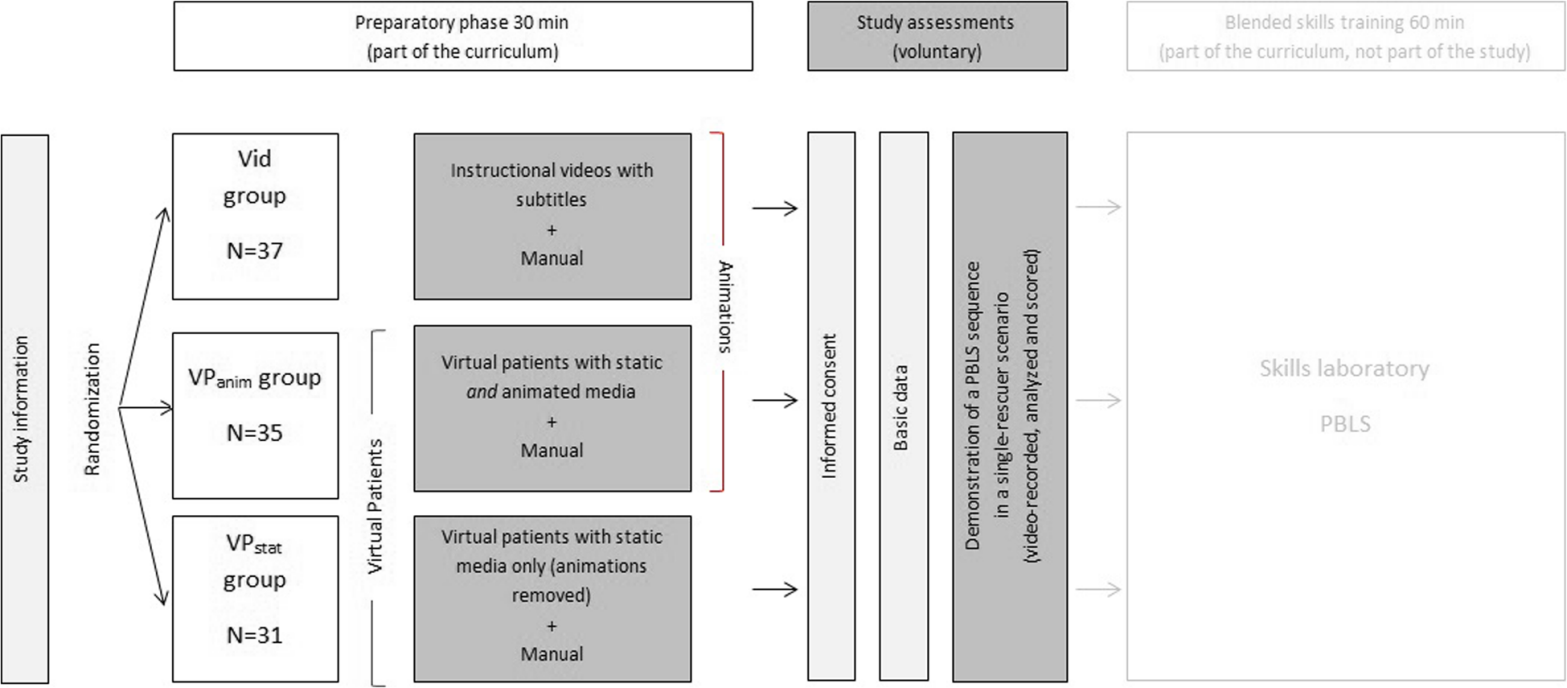
Study design. The video group (Vid) received instructional, animated videos, the animated VP group (VP_anim_) received VP containing animated components such as interactive questions and animated media, and the static VP group (VP_stat_) received VP containing interactive questions and *only* static media. *N* = 103 participants were included and randomized to the study groups

In this prospective study, we used a three-group, randomized trial design, as shown in Fig. 1. All participants received general information about the study course and were randomized to one of the following groups:

- Video group (Vid): self-instruction with videos (infant and toddler) on PBLS.
- VP with animations (VP_anim_): self-instruction with VP (infant and toddler) on PBLS containing interactive questions and graphics, static media (pictures) *and* animations (video clips).
- VP with only static media (VP_stat_): self-instruction with VP (infant and toddler) on PBLS containing interactive questions and graphics, and *only* static media.

Blocked Randomization was used with block sizes of 9 to 12 (depending on rotation sizes) and an allocation ratio of 1:1:1. Participants were alphabetically grouped by the pediatric course administration for the curricular PBLS courses, and each group (block) received a different preparation method in a sequence predefined by the study conductors, but concealed to participants and course administration. Groups were not stratified for any demographic data which was assessed later.

Furthermore, all participants received a manual explaining the algorithm and steps of PBLS including flow charts. For self-instruction, a preparatory phase of 30 min was given with either VP work-up or watching the instructional videos. Written informed consent to participate in the study was obtained as well as basic demographic data. Participants then demonstrated a whole sequence of PBLS as described below. Demonstrations were videotaped, analyzed and scored for adherence to guideline recommendations [25] in terms of algorithm, temporal demands and procedural quality as described below.

### Virtual patients and video instruction development

**Fig. 2:**
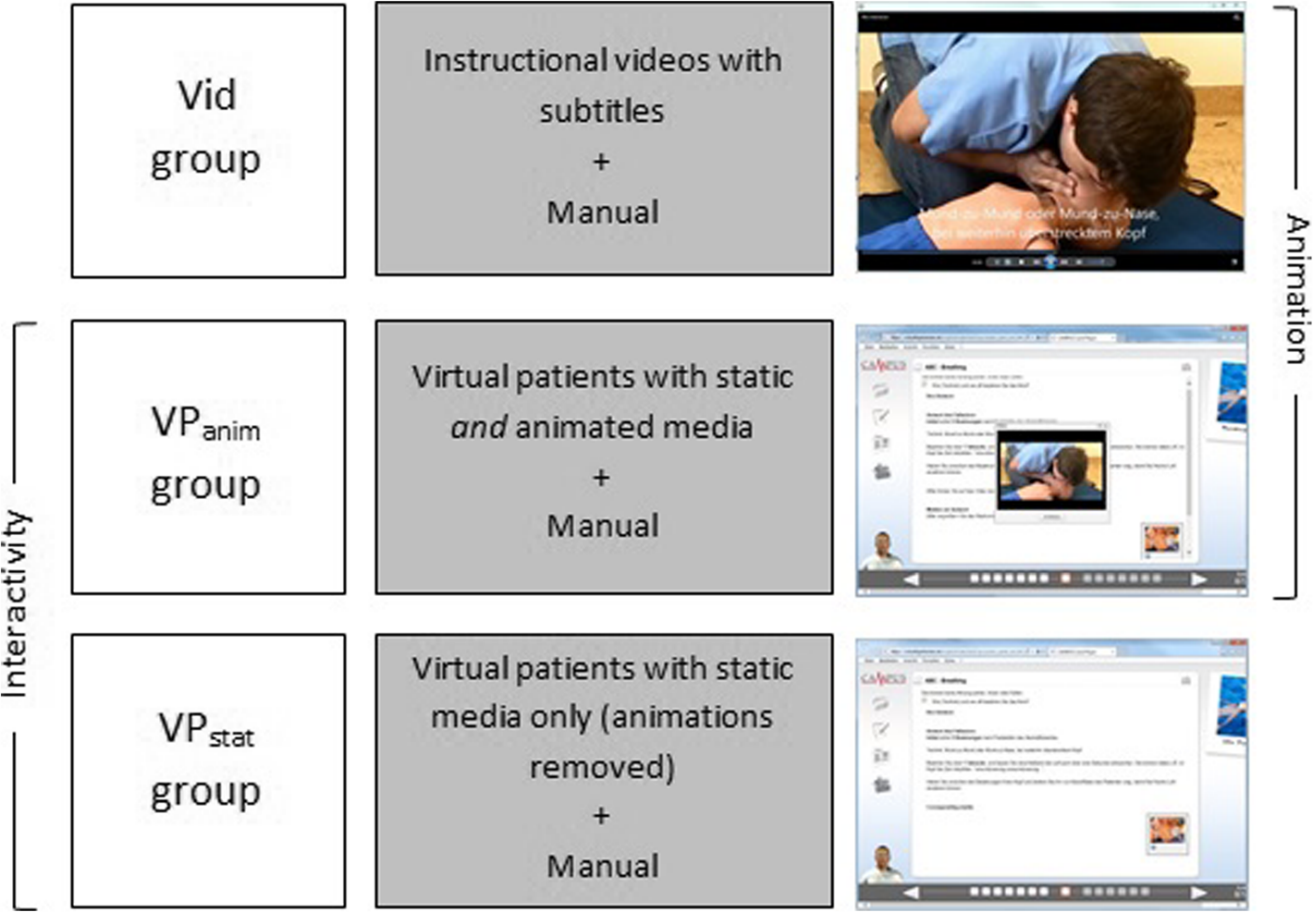
Study groups. Similarities and differences of formats in the study groups on the example scene ‘initial 5 rescue breaths’. Animations are used in groups Vid and VP_anim_, while a case-based, interactive learning environment is provided by groups VP_anim_ and VP_stat_ in the format of a virtual patient

VP were developed using CAMPUS software [26] and according to published design criteria [19]. The points of origin were existing VP on PBLS implemented in the curricular skills training that our study group described earlier [24]. These VP rely on interactive questions and interactive graphics, and also include media elements such as static pictures and video clips of complete PBLS sequences from approaching the patient to making an emergency phone call after 1 min of PBLS at the end. VP’ contents are designed according to current PBLS guidelines [25]; they have been modified as follows for this study, see also Fig. 2:

For group VP_stat_, video sequences were removed serving as quasi-control for the effects of animations.

For group VP_anim_, video sequences were kept and supplemented by numerous additional video cutouts of each particular procedural step of the PBLS algorithm that enriched the corresponding VP slides in the sense of spatial contiguity [27]. This design principle assumes deeper learning when textual description and corresponding media are presented close together or integrated, e.g. a video clip of opening the airway directly next to its written description.

Group Vid received instructional videos instead that were composed of all single video sequences used in group VP_anim_. These were subtitled with all comments taken from those VP. In addition, the whole PBLS sequences were shown without comment in real-time at the beginning and again at the end of the videos. Group Vid served as quasi-control for the interactivity provided by VP.

### Evaluation procedure

Before the preparatory phase, general information about the study — but not about its design, goals or hypotheses — was given to participants. Students received individual access to either VP or instructional videos by randomized access codes on stationary computers. They were strongly advised to work up each respective preparation material at least twice during the preparatory phase lasting 30 min. After this, they could choose to participate in the study and then, after providing informed consent and their demographic data, demonstrated PBLS separately and unobserved by their colleagues in a simple single-rescuer scenario. Participants were instructed to demonstrate the PBLS sequence from approaching the child, one minute of CPR, to the emergency phone call afterwards according to guideline recommendations [25]. They were encouraged to act realistically; interruptions or further inquiries were not allowed during the demonstrations. Manikins by Simulaids Inc., Saugerties NY, USA, were used as patient simulators of a toddler.

Delivery of study activities and data acquisition including video-recordings were performed by third persons not aware of group allocation or study hypotheses.

### Instruments and measures

Participants provided their demographic data (gender, age and whether they had participated in PBLS trainings before). Especially prior PBLS training could be one of the biggest confounders in the allocation of participants as described earlier [23]. As primary outcome measures, video assessors gave overall competency ratings on each participant’s performance whether it was competent or not. For group comparisons, only ‘competent’ ratings in consent by both raters were included into further analysis. The videotaped PBLS sequences were scored in the domains of adherence to the correct algorithm, adherence to temporal demands and procedural quality as secondary measures using rating schemes developed by our research group (for scoring forms in detail, see appendices of Lehmann et al. [23]). Adherence to the correct algorithm was scored assessing each particular procedural step as well as its correct order. Temporal demands derive from specific temporal recommendations in the guidelines such as a defined chest compression rate of at least 100 but not exceeding 120 compressions per minute. Benchmark time periods for particular procedural steps as well as the total time of the sequence were determined, respectively, and participants’ actual deviations were scored. Two group-blinded video raters evaluated the demonstrated quality of procedural steps of the PBLS algorithm that are described in the guidelines. Each item here was scored independently if performed in a correct, partially incorrect or considerably incorrect manner (or not rateable, respectively). Items were not weighted and average scores were used for further analyses. Algorithm and temporal aspects were not taken into account for procedural quality scorings, as these were measured and scored separately.

### Rater selection and training

Two video raters located external to the study location scored videotaped PBLS demonstrations without being made aware of participants’ group allocations. Both were experts in the field of PBLS and simulation-based medical education. Rater training included a review of the case content and objectives, and an introduction to the rating schemes followed by discussion and calibration. Benchmark videos from the origin VP were also provided to calibrate expectations. Both raters are also co-authors of this study (SK, HMB).

### Statistical analyses

Based on a prior study by our group [23] we estimated an effect of 20% ‘competent’ ratings in the group VP_stat_, and we assumed an effect of 50% ratings as ‘competent’ in those groups using animations as primary outcome measure. For this effect size, assuming an alpha of 0.05, one-sided testing and a statistical power of 80%, a sample size of 31 per group was calculated. Similarly, other authors recommend a minimum of 30 individuals per group for such experimental and causal comparative studies [28].

Continuous variables are given as means and standard deviation, categorical (dichotomous) data by absolute numbers and percentages per group. For statistical comparisons of the participants’ demographic data, chi-square tests were conducted for gender and previous PBLS training, as well as a one-factor analysis of variance (ANOVA) with the between-subject factor ‘Group’ (Vid vs. VP_anim_ vs. VP_stat_) and the dependent variable ‘Age’. To determine PBLS performance in secondary outcome measures, data were previously checked for normal distribution using the Kolmogorov-Smirnov test. If normal distributions could be assumed, an one-factor multivariate analysis of variance (MANOVA) with the between-group factor ‘Group’ (Vid vs. VP_anim_ vs. VP_stat_) and the within-subject factor ‘Domain’ (adherence to correct algorithm, adherence to temporal demands, procedural quality) was conducted. A statistically significant MANOVA was followed by individual analysis of variance (ANOVA), and LSD post hoc tests where appropriate. Bonferroni corrections were conducted for all multiple comparisons. Effect sizes (Cohen’s *d*) were calculated to support interpretation of group differences according to [29, 30]. To compare overall competency assessments of the video raters, a chi-square test was conducted. Interrater reliability of the procedural quality ratings was calculated using the Intraclass Correlation coefficient (ICC 2,k). Data were analyzed using IBM SPSS Statistics Version 24 (IBM Corporation, Armonk NY, USA). Secondary outcome measures were two-sided and differences were considered significant when *p* < 0.05.

## Results

### Demographic data

Of 136 students rotating through pediatrics in the study period, *N* = 103 (75.7%) agreed to participate in this study and were randomized to groups Vid (*N* = 37), VP_anim_ (*N* = 35) and VP_stat_ (*N* = 31). The three study groups did not differ in distribution of gender, previous PBLS training nor age (Table 1).

**Table 1:** Basic data of participants

	Video group (Vid) *N* = 37		Animated VP group (VP_anim_) *N* = 35		Static VP group (VP_stat_) *N* = 31		
	*N*	%	*N*	%	*N*	%	
Male	24	64.9	21	60.0	17	54.8	*p* = 0.70^1^
Female	13	35.1	14	40.0	14	45.2	
Had previous PBLS training	8	21.6	3	8.6	4	12.9	*p* = 0.28^1^
	mean ± SD		mean ± SD		mean ± SD		
Age (years)	25.4 ± 2.8		24.8 ± 2.2		25.1 ± 2.9		*p* = 0.63^2^

^1^χ2 test, ^2^ ANOVA, Allocation of gender and previous PBLS training as *N* and percentages, and for age as mean and standard deviation (*SD*)

### Domain differences between study groups

In the MANOVA examining the association between study group and domain, their interaction was strongly significant with *F*(6,198) = 8.081, *p* < 0.001. For detailed domain results of adherence to the correct algorithm, adherence to temporal demands and procedural quality, see Table 2.

**Table 2:** Scoring results in the domains of algorithm, temporal demands and procedural quality

Domain	Video group (Vid) *N* = 37	Animated VP group (VP_anim_) *N* = 35	Static VP group (VP_stat_) *N* = 31	VP_anim_ vs. Vid	VP_stat_ vs. Vid	VP_anim_ vs. VP_stat_
	mean ± SD	mean ± SD	mean ± SD			
Adherence to correct algorithm	88.7 ± 10.3	93.3 ± 6.7	90.3 ± 10.5	ns^1^	ns^1^	ns^1^
				*d* = 0.53	*d* = 0.15	*d* = 0.34
Adherence to temporal demands	64.5 ± 26.3	50.7 ± 25.7	23.8 ± 21.0	*p* = 0.06^2^	***p*** **< 0.001**^2^	***p*** **< 0.001**^2^
				*d* = − 0.53	*d* = − 1.71	*d* = 1.15
Procedural quality	79.5 ± 12.3	82.0 ± 11.9	73.2 ± 11.9	*p* = 1.00^2^	*p* = 0.11^2^	***p*** **= 0.01**^2^
				*d* = 0.21	*d* = − 0.52	*d* = 0.74

^1^ANOVA not significant,^2^ Post hoc test, Results as mean and standard deviation (*SD*) in percentages of achievable scores from 0 to 100 (maximum score). Statistically significant results between two respective groups are indicated in bold

### Adherence to the correct algorithm

ANOVA did not indicate statistical significance for the domain ‘adherence to correct algorithm’ with *F*(2,100) = 2.246, *p* = 0.111. No post hoc tests were conducted.

### Adherence to temporal demands

Statistical significant differences were found between groups for adherence to temporal demands with *F*(2,100) = 23.540, *p* < 0.001. Post hoc tests revealed superior and highly significant differences for both groups using animated media (group Vid and VP_anim_) compared with group VP_stat_. Here, very large effect sizes were found. Of both groups using animations, group Vid showed the best adherence to temporal demands compared with group VP_anim_ with a medium-sized effect, although not significant when compared with each other.

As an example of temporal differences between groups, Table 3 shows measured total time of PBLS sequences starting from approaching the patient and ending when the participant interrupted CPR for an emergency call. A benchmark period of 80 s was determined [23]. ANOVA was *F*(2,100) = 29.424, *p* < 0.001; all three groups showed highly significant differences between each other in post hoc tests. Effect sizes can be considered large or very large in comparison with group Vid— the only group performing close to the benchmark — against both VP groups (groups VP_anim_ and VP_stat_). Also, the VP group using animated media (group VP_anim_) showed significantly improved total PBLS time with a large effect size as compared with the VP group using only static media (group VP_stat_), which had the poorest performance.

**Table 3:** Total time of PBLS sequence (part of ‘adherence to temporal demands’ domain)

	Video group (Vid) *N* = 37	Animated VP group (VP_anim_) *N* = 35	Static VP group (VP_stat_) *N* = 31	VP_anim_ vs. Vid	VP_stat_ vs. Vid	VP_anim_ vs. VP_stat_
	mean ± SD	mean ± SD	mean ± SD			
Total time of PBLS sequence (seconds)	84.6 ± 10.4	98.8 ± 16.8	120.9 ± 28.6	***p*** **= 0.008**^1^	***p*** **< 0.001**^1^	***p*** **< 0.001**^1^
				*d* = 1.02	*d* = 1.69	*d* = − 0.94

^1^Post hoc test, Results as mean total time and standard deviation (SD) for a PBLS sequence, in seconds. Benchmark was determined as 80 s. Statistically significant results between two respective groups are indicated in bold

### Procedural quality

Differences in procedural quality between groups reached statistical significance with *F*(2,100) = 4.537, *p* = 0.013. Group Vid and group VP_anim_ did not differ, but post hoc tests showed a significant superior performance by the VP group using animated media (group VP_anim_) compared with the VP group that used only static media (group VP_stat_). This effect size was medium to large.

### Overall competency rating

The overall competency rating of the three study groups differed highly significant with χ^2^ = 14.366, *p* = 0.001 (Table 4). The VP group using animated media (group VP_anim_) received distinctly more ‘competent’ ratings than the video group (group Vid), which on her part performed better than the VP group using only static media (group VP_stat_).

**Table 4:** Overall competency rating

	Video group (Vid) *N* = 37		Animated VP group (VP_anim_) *N* = 35		Static VP group (VP_stat_) *N* = 31		
	*N*	%	*N*	%	*N*	%	
Performance rated ‘competent’ in consent	16	43.2	23	65.7	6	19.4	***p =*** **0.001**^1^

^1^χ2 test, Number of participants rated ‘competent’ in the three respective groups as *N* and percentages. Statistically significant results between respective groups are indicated in bold

### Interrater reliability

The intraclass correlation coefficient of interrater reliability was 0.822 between the two video assessors. This is an expected correlation between any two randomly selected raters on the same individual performance.

## Discussion

PBLS is a complex clinical procedure. We argue that a differentiated multidimensional approach is necessary to assess learning progress and identify elements that facilitate its acquisition and retention. Order of action, pace of execution and quality need to be regarded separately when comparing differing methods of teaching and their impact on the domains of procedural learning. Overall competency ratings by experienced assessors do integrate and complement such dimensions by weighting in consideration of clinical requirements. Here, we analyze the effect of two main elements of instructional methods on successful competence acquisition. First, animation as provided by video instruction or within VP, and second, interactivity as provided by VP.

In terms of domain adherence to the algorithm, we found no significant difference between the groups. All three formats prepared the students well for applying the correct algorithm. In our previous study, a significant benefit was found for interactive and multimedia preparation with VP compared with passive, paper-based self-instruction when assessing algorithm adherence [23]. In this study, a comparable level of algorithm adherence was also reached with instructional videos.

In terms of temporal demands, formats using animation (groups Vid and VP_anim_) were superior. Applying only static media led to significantly poorer results. Videos or embedded video clips within VP effectively convey dynamic aspects of the activity to the learner. This requires less cognitive effort to process than text description and static media [4, 5, 31]. Having seen PBLS in real-time video beforehand might have set an internal framework of the procedural flow that textual and graphic description cannot provide, at least among learners with enough so-called spatial ability for advanced learning [31]. Study participants who had already undergone CPR training (in adults) as part of the medical curriculum cannot be considered as beginners. Video-instruction — providing the lowest cognitive load — seemed best for emphasizing the value of an adequate pace in performing PBLS, although this was not addressed explicitly.

The procedural quality of PBLS was also significantly superior in the animated media compared with the static media VP group, with equivalent high ratings in the video group. The combination of animation and interactivity seems best for instruction in correct handling of particular algorithm steps; interactivity alone had an inferior effect if not provided together with animated media or video clips, respectively.

The global rating of overall competency was strongest in discriminating differential effects of the three investigated formats; 65% of the participants using VP with animations (videos) were judged competent after that preparation, as compared with 43% after (passive) video-instruction and only 19% using static-media only VP. Using animations seems to facilitate the acquisition of PBLS competency. Other approaches using videos also showed equal or superior skill acquisition and retention compared with traditional instructor-led training [2, 3, 32–35]. In contrast, poorer CPR performances were reported as well after video self-instruction compared with traditional methods [36]. In this context, Mpotos et al. indicated that additional feedback on learners’ performances is necessary to achieve acceptable CPR skills after self-led video instruction [37]. The best performance in our study was shown by the group VP_anim_ which is in line with these findings, as they actively involve the learner with an adequate level of cognitive load for optimal learning [31] *and* provision of animation for unburdened uptake and processing of learning content.

VP seem to increase the level of realism and may set an emotionally activating stimulus by providing a case-based environment, which has been shown to strengthen retention of BLS skills [38]. By adjusting the learning process, the interactivity and feedback of VP likely represent their most important features [19, 39]. The presented content including media can be tailored in a VP for an optimal application of research-based principles such as the spatial contiguity that affects learning [27]. We found a competency rate of only 19% after preparation with interactive VP that were not using animated media similar to our former study [23], which indeed included one video clip but was not conducted in a controlled environment like that in the present study. In the present study, the rate of competency was increased to almost two-thirds of all participants practicing with improved and animation-enriched VP. Here, competent PBLS performance was demonstrated by the majority of students afterwards without having had hands-on training (yet).

Blended learning approaches have revealed ambiguous results: Thorne et al. reported equivalent learning outcomes between a conventional course and a blended course with e-learning and a shortened face-to-face phase [8]. Perkins et al. reported slightly lower pass rates in practical assessments when substituting parts of the training by e-learning [40]. The challenge is to find the optimal blending of both methods [9, 41]. Doing so might also have a positive effect on skill retention, as a spaced format to teach resuscitation is believed to be favorable compared with a massed training [42]. For PBLS training, our research group showed superior performance after working on VP, and this improvement was still significant *after* hands-on training sessions [23]. In that former study, VP not only provided better instruction for, but also an increase in the effectiveness of a subsequent tutor-led training.

After merging all evidence, our results show that VP provide an appropriate preparation tool for blending with traditional resuscitation training. They allow integration of animations and other media that are particularly effective in conveying dynamic aspects of procedures. Because they require interaction and active learning, VP contribute to improved cognitive frameworks for the tasks to be learned. The learning process can be optimized by providing feedback and practice in case-based scenarios. Blended with a consecutive hands-on training, a spaced format also contributes to optimized and substantial learning.

### Limitations

This study has several limitations. Although this study is sufficiently powered, randomization potentially may not have eliminated all existing differences in proficiencies and skills among participants, or differing conscientiousness in preparations, due to its limited sample size. A cross-over design using two more clinical skills could have considerably improved statistical evidence, but the choice on similar complex procedures with established and multidimensional (algorithm, temporal, quality) assessment forms is limited. Also the use of non-validated assessment tools for PBLS performance is a limitation although their face validity is high. Crucial issues like transferability to clinical practice and sustained retention of skills over time were not addressed and should be further investigated. Subjective perceptions of participants assessed with questionnaires or focus groups were not addressed in this study for triangulation.

## Conclusions

Animations, especially videos, are important components of instruction resulting in improved pace and procedural quality of PBLS. While video instruction alone leads to suboptimal performance quality, the combination of animations and interactive learning by detailed VP facilitates optimal reflective skill acquisition. In addition, VP with embedded video clips can best address demands in different dimensions (algorithm, pace, quality), and allow flexible access and blending with hands-on training.

## Abbreviations

AED: Automated external defibrillator
BLS: Basic life support
CPR: Cardiopulmonary resuscitation
PBLS: Pediatric basic life support
VP: Virtual patient(s)
